# HLA‐F: A Non‐Classical Gene With Growing Interest

**DOI:** 10.1111/tan.70547

**Published:** 2026-01-15

**Authors:** Nina Grünen, Liane Hey, Yannik Busch, Marco Schäfer, Ilias Doxiadis, Anne Schweizer, Wolfgang Peter

**Affiliations:** ^1^ HLA Laboratory of the Stefan Morsch Stiftung Birkenfeld Germany; ^2^ Umwelt‐Campus Birkenfeld Birkenfeld Germany; ^3^ Institute for Transfusion Medicine University Hospital Leipzig Leipzig Germany

## Abstract

The HLA‐F is a non‐classical HLA class I gene that belongs to the class Ib major histocompatibility complex (MHC) molecules. It is distinguished in the literature from its classical MHC class Ia counterparts by a minor frequency of polymorphisms. The exact function of HLA‐F remains unknown, but recent publications emphasise the essential immunological role of this gene in infectious diseases, cancer research, organ transplantation and autoimmune diseases. The rapidly evolving sequencing techniques within the last years allow the accession of larger genomic regions at reasonable costs. In this regard we developed a long‐range PCR assay, covering the entire HLA‐F gene, including the flanking untranslated regions (UTRs). According to the positions of the 5′ and 3′ amplification primers, the PCR amplicon has a total length of 3.8 kb. After verification of our in‐house developed LR‐PCR with pre‐typed cell‐line derived DNA‐samples from International HLA Reference Standards (IHWG), we analysed a randomly selected cohort of 763 DNA samples from the Stefan Morsch Stiftung stem cell donor registry on a MiSeq next generation sequencing (NGS) platform. This HLA‐F genotyping project revealed so far unpublished data regarding the allele frequency distribution pattern as well as several new allelic variations, not listed yet in the IPD‐IMGT/HLA Database. Prior to submission, all novel alleles were confirmed with Oxford Nanopore sequencing. Linkage disequilibrium between HLA‐F and its neighbouring loci HLA‐A and HLA‐E was also assessed.

## 1 Introduction

### 1.1 Structural and Functional Characteristics of HLA‐F

HLA‐F is a non‐classical MHC class I molecule encoded on chromosome 6, spanning approximately 3.5 kb. The protein consists of 346 amino acids and has a molecular weight of about 39 kDa. Structurally, the HLA‐F gene shares its general organisation with other HLA class I genes, comprising eight exons interspersed with non‐coding introns. These exons encode a signal peptide (exon 1), three extracellular domains (α1, α2, α3; exons 2–4), a transmembrane region (exon 5), and a cytoplasmic tail (exon 6–8).

A notable distinction of HLA‐F is that exon 7 remains untranslated. As a result, the cytoplasmic tail of HLA‐F is approximately 2 kDa shorter compared to that of classical HLA class I molecules. This structural truncation influences intracellular signalling and protein trafficking. Furthermore, variability in the translated cytoplasmic region gives rise to multiple HLA‐F isoforms with potentially distinct functional properties.

In addition, the 3′‐untranslated region (UTR) of HLA‐F differs substantially from those of other HLA class I genes, which may influence mRNA stability, localisation, and translation efficiency. These unique structural and regulatory features underscore the specialised function of HLA‐F in immune modulation and tolerance [1].

**FIGURE 1:**
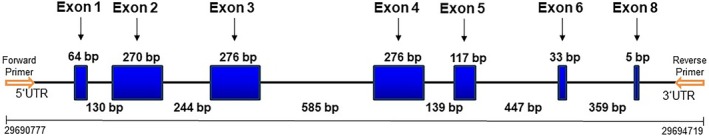
Schematic representation of the HLA‐F gene structure, showing exons 1–6 and 8, along with intronic regions. The relative sizes of exons and introns are indicated. Exon 7 is omitted due to its classification as a pseudoexon. The amplified region with used forward and reverse primer is implied through the orange arrows in UTR region (Reference Genome Build: 37.1).

A schematic overview of the complete gene structure of HLA‐F is provided in Figure 1.

HLA‐F has distinct intracellular and surface expression patterns. Under resting conditions, it is predominantly localised intracellularly, particularly in peripheral blood B cells and certain tissues such as foetal liver, tonsils, thymus, and various tumour cells. Upon cellular activation, HLA‐F translocates to the cell surface, where it is expressed on activated immune cells (including B, T, and NK cells), virus‐infected cells, and specific tissues like pancreatic islets and invasive placental cells, suggesting a regulatory role in immune responses. Its mRNA transcription and surface expression can be induced in activated CD4^+^ T cells by interleukin‐2 (IL‐2), as well as by chemical agents like phorbol 12‐myristate 13‐acetate (PMA) and ionomycin [2]. Unlike classical HLA class I molecules, HLA‐F does not require tapasin or TAP for endoplasmic reticulum (ER) transport but instead depends on a C‐terminal valine and RxR (arginine‐any‐arginine) motif, which serves as an ER retention signal regulating its export [3].

Functionally, HLA‐F exists in two conformations: A β₂‐microglobulin (β₂m)‐associated, peptide‐loaded trimeric complex and an open conformer (OC), lacking both β₂m and peptide. The peptide‐bound form can present peptides of unconventional lengths, whereas the open conformer can interact with immune receptors such as KIR3DS1 and KIR3DL2 on natural killer (NK) cells.

Among known immune receptors, the inhibitory leukocyte immunoglobulin‐like receptors ILT2 (LILRB1) and ILT4 (LILRB2) specifically recognise the peptide‐bound HLA‐F:β_2_m complex, but not the open conformer. ILT2 and ILT4 are broadly expressed across various immune cells, including NK cells, T cells, dendritic cells, B cells, and monocytes, and they signal through immunoreceptor tyrosine‐based inhibition motifs (ITIMs) in their cytoplasmic domains.

In contrast, activating killer‐cell immunoglobulin‐like receptors (KIRs) such as KIR3DS1 and KIR3DL2 primarily bind to HLA‐F in its open conformer form. KIR3DS1 exhibits high affinity for the open conformer, while KIR3DL2 binds with lower affinity. This functional distinction underscores the importance of HLA‐F conformation in modulating immune responses, with ILT2 and ILT4 playing central roles in mediating immunosuppressive signals specifically through recognition of peptide‐loaded HLA‐F complexes.

These findings highlight a sophisticated layer of immune regulation in which HLA‐F's structural state governs its interaction with inhibitory and activating receptors, suggesting a specialised role for ILT2 and ILT4 in immune tolerance and homeostasis [1].

### 1.2 Variation and Disease Links

While the classical HLA class I genes have been studied for many years due to their importance in transplantation, autoimmunity and cancer, the functional role of the non‐classical class I genes still remains cryptic and hardly understood. The recent access to high‐throughput and cost‐efficient sequencing methods drew more attention to those genes and increased the number of studies.

However, the exact function of HLA‐F molecules remains unknown. There is autoimmunity by signalling through NK‐cell receptors (NKR) [4]. Clinical and experimental studies have shown the implication of non‐classical HLA class I in several diseases such as inflammatory or autoimmune diseases [1], cancer [5], and viral infections [1]. The expression of HLA‐F has been associated with gastric or breast cancer [6], coronary artery diseases [7], and encephalitis virus [8], as well as with chronic HBV infection.

With 126 different alleles registered at the IPD‐IMGT/HLA Database (Release 3.62.0, 2025‐10‐08) encoding for 30 different proteins, HLA‐F is often rated as a locus with a low degree of polymorphism. Indeed, the gene seems to be less polymorphic compared to other HLA genes. According to Pan et al., one has to consider the lack of information on HLA‐F polymorphism rates within the ethnically diverse human populations [9] or the allelic variations among human populations not studied so far. Pyo et al. were able to not only confirm several previously reported coding sequence variants in their analysis but also to identify several new allelic variants and define extensive variations in the noncoding intron and flanking UTR sequences, showing that the HLA‐F gene is not fully characterised yet [10]. This supports the assumption that the gene has a higher allelic polymorphism than described and that a closer examination of the non‐classical gene might generate new insights in immune response.

Assays for high‐throughput genotyping of the classical HLA class I genes are now commercially available, but the lack of widely used assays for the non‐classical loci induced us to develop an NGS method to obtain reliable sequence information covering the entire HLA‐F gene including the flanking UTRs.

## 2 Methods

### 2.1 Sample Selection and DNA Preparation

All samples genotyped in this study were recruited from the Stefan Morsch Stiftung (a German foundation) as potential stem cell donors between 2017 and 2018. All participants provided their informed consent. The majority of the samples are of European origin; however, the exact ethnicity of the donors could not be determined due to the anonymised nature of the sample handling. Genomic DNA was extracted from peripheral blood leukocytes and saliva samples (Oracollect, Canada) using the chemagen DNA‐Isolations Kit on the chemagic MSM1 device (Baesweiler, Germany). The quality and quantity of the extracted DNA were assessed by measuring the absorbance value ratios at 260 and 280 nm. DNA working solutions were adjusted to 10–20 ng/μL.

### 2.2 HLA‐F Primer Design

The design of the corresponding long‐range PCR primers to target the entire HLA‐F gene was initiated with 10 different primers located either in the upstream or the downstream untranslated region. The primer binding sites were chosen in compliance with the reference sequences from the IPD‐IMGT/HLA Database (https://www.ebi.ac.uk/ipd/imgt/hla/align.html) and the 1000 Genomes Project (https://www.ncbi.nlm.nih.gov/variation/tools/1000genomes), considering all likely appearing sequence variants such as SNPs or Indels. A GC content in the range of 30%–70% and a salt adjusted melting temperature of approx. 65°C were the physical guidelines for the primer design. These parameters were checked using Oligo Calc (http://biotools.nubic.northwestern.edu/OligoCalc.html), an oligonucleotide properties calculator, which was also applied to avoid primer binding interferences caused by hairpin and dimer formations. The amplicon size and locus specificity of all the designed primers were calculated using the online tools NCBI BLAST (https://blast.ncbi.nlm.nih.gov/Blast.cgi) and SNP check (https://genetools.org/SNPCheck/snpcheck.htm). The sequences of the finally chosen forward and reverse primer are available upon request to the corresponding author under a non‐disclosure agreement.

### 2.3 HLA‐F Long Range PCR

Initial performance testing of the designed primers was conducted using 14 pretyped DNA samples with available results for HLA‐F, obtained from the International Histocompatibility Working Group (IHWG) (Table S1). These samples represent the most common HLA‐F alleles in European populations. All primer combinations were tested across these cell lines to evaluate amplification specificity and efficiency.

To assess primer robustness under more challenging conditions, additional testing was performed using DNA isolated from saliva samples, as both blood‐ and saliva‐derived DNA are routinely used in the laboratory of the Stefan Morsch Stiftung. Given the typically lower concentration and quality of saliva DNA, this served as a more stringent condition for evaluating PCR performance. For each primer pair, PCR was performed on eight saliva DNA samples. While non‐specific products were rarely observed in agarose gel electrophoresis, several primer combinations resulted in amplification dropouts or inconsistent performance.

Multiple PCR conditions and thermocycling programs were tested during this optimisation phase. The final optimised setup, which provided the highest specificity and amplification success, was used for all subsequent experiments.

The conclusive long‐range PCR setup contained 10 μL GoTaq Long PCR Master 2× Mix (Promega, Walldorf), 1 μL (5 μM) each of the forward and reverse HLA‐F specific primer, and 2 μL of genomic DNA (20–40 ng), with nuclease‐free water added to a total volume of 20 μL. PCR amplification was performed on an ABI9700 thermocycler (Applied Biosystems) with the following conditions: initial denaturation at 95°C for 2 min; 40 cycles of denaturation at 98°C for 15 s, annealing at 65°C for 15 s, and elongation at 68°C for 2 min; followed by a final extension at 68°C for 10 min.

After PCR amplification, the amplicons were diluted by adding 30 μL water to the initial reaction mixture of 20 μL. As amplification control, an aliquot of 4 μL was mixed with 2 μL loading buffer and separated for 1 h on a 1% TAE agarose gel at 5 V/cm gel length.

Only samples revealing a separation pattern with a clear and definite band in the calculated size range, lacking unspecific side products, were considered valid and used for the subsequent PCR pool containing 5 μL amplicon per locus.

### 2.4 Next Generation Sequencing (NGS) on a Short‐Read Platform (MiSeq Illumina)

The library preparation of the HLA‐F long‐range PCR products started with a 0.7× AMPure bead purification step on a Biomek NXp automated workstation (Beckman Coulter, Krefeld, Germany). The following library preparation steps such as fragmentation, end repair, adapter ligation, and the index PCR were performed according to the instructions of the manufacturer (NEB, Frankfurt).

To monitor the library efficiency we performed a SybrGreen fluorescence measurement using a GloMax‐Multi+ reader (Promega, Walldorf) before proceeding with the fragment size selection. To enrich library fragments ranging from 600 up to 800 bp we separated the library fragments on a 1,6% TAE agarose gel at 5 V/cm for 40 min. A thin slice in between the migration range of the laterally applied size markers was excised and weighted for gel extraction with the GeneJET Gel Extraction Kit (ThermoFisher Scientific, Darmstadt). To estimate the concentration of the size‐selected library eluate, a quantitative PCR was performed with a KAPA Library Quantification Kit for Illumina platforms (Roche, Mannheim) on a QuantStudio 5 Real‐Time PCR System (ThermoFisher Scientific, Darmstadt). The size‐selected library samples were diluted to a final concentration of 12 pM and a total amount of 600 μL was added into the cartridge of a MiSeq platform (Illumina, Berlin) with 500 V2 chemistry, to perform DNA sequencing.

HLA‐F allele assignment was done using the software tool NGSengine Version 2.9.1 (GenDx, Utrecht), working with sequencing‐based typing (SBT) for the data generated by NGS. Validation of the typing results was done with a second software product called HLA Twin Version 2.51 (Omixon, Budapest), working with two independent algorithms—Consensus Genotyping and Statistical Genotyping. IPD‐IMGT/HLA Database Version 3.33 (2018‐07) was used in both cases as reference database. Exon mismatch(es) between reference sequence and consensus sequence are highlighted as a novelty by the software, named by the most related reference sequence followed by a ‘#1’. For example, the HLA‐F*01:01:01:09 novelty (later assigned as F*01:10) was identified as HLA‐F*01:01:01:09#1 at this point of routine analysis.

HLA‐A and HLA‐E typing was included in the analysis for the calculation of linkage disequilibrium in subsequent steps, using the same methodology reported above. The library preparation of the HLA‐A and HLA‐E long‐range PCR products has already been established as part of the routine laboratory procedures at the Stefan Morsch Stiftung.

### 2.5 HLA‐F Long Read Sequencing (MinIon, Oxford Nanopore Technology)

For samples in which short‐read sequencing data indicated the presence of a novel nucleotide substitution within exon regions of the HLA‐F gene, the locus was selectively reamplified in an independent PCR reaction. The resulting amplicons were then analysed using the long‐read sequencing platform from Oxford Nanopore Technologies (ONT) (Oxford, England).

Library preparation was performed according to the ONT protocol *Native Barcoding of Genomic DNA* (Kits EXP‐NBD196 and SQK‐LSK109). Initially, DNA fragments underwent end‐repair and dA‐tailing using the NEBNext Ultra Kit (New England Biolabs, Frankfurt). Sample‐specific barcodes were then ligated using the Native Barcoding Kit EXP‐NBD196. Following barcoding, samples were pooled, and sequencing adapters containing leader sequences and motor proteins were ligated using the Ligation Sequencing Kit SQK‐LSK109. Sequencing was conducted on an ONT MinION platform equipped with an R9.4.1 flow cell over a 24‐h run, yielding approximately 2.17 million reads and a total of 4.44 Gb of passed bases. Basecalling and demultiplexing were performed post‐run with Guppy v4.0.11 in high‐accuracy mode, applying a minimum quality threshold of Q7 and enabling adapter trimming.

Run quality and sequencing performance were assessed using NanoPlot (https://doi.org/10.1093/bioinformatics/btad311), an open‐source tool for visualisation and quality control of nanopore sequencing data. Sequence pre‐processing was carried out with NanoFilt (https://doi.org/10.1093/bioinformatics/bty149), applying filtering parameters informed by run‐specific metrics, specifically a minimum read length of 4 kb and a minimum quality score of Q12. Filtered reads were subsequently analysed with NGSengine v2.25. Within the software, the alignment algorithm was set to ‘Cluster’, and quality trimming was disabled. Genotyping was performed against the IPD‐IMGT/HLA Database Version 3.47 (2022‐01). Fully phased sequences, along with corresponding gene reports, were exported using the ‘Prepare for submission’ function implemented in NGSengine.

### 2.6 Statistical Analysis

The allele frequency of 763 samples typed for HLA‐F was obtained by direct counting. Genotype resolution discrepancies between the two different NGS typing software tools HLA Twin and NGSengine, observed in approximately 2% of cases and limited to the fourth field, were excluded from the summary. These differences are primarily due to the distinct analytical approaches employed by the two software solutions.

Hardy–Weinberg equilibrium (HWE) was assessed as part of routine quality control procedure for the genotypic data. Expected genotype frequencies were calculated from allele frequencies using the equation *p* ^2^ + 2*pq* + *q* ^2^ = 1, where *p* and *q* represent the frequencies of the two alleles. Loci showing significant deviation from HWE (*p* < 0.05) were excluded from further analysis to reduce the risk of genotyping errors or population stratification.

Pairwise linkage disequlibrium (*D*) was calculated as described before by Hill et al. [11]. As no haplotype information between the pairwise loci was available, the haplotype frequencies were estimated employing the expectation–maximisation (EM) method as suggested by Slatkin et al. [12]. Calculations were performed in R utilising the Haplo.Stats R package (10.32614/CRAN.package.haplo.stats). Briefly, the EM algorithm for unphased diploid genotyping data infers underlying haplotype or allele‐frequency parameters by iteratively alternating between computing expected genotype–component probabilities and maximising the likelihood of those expectations.

In addition to the raw linkage disequilibrium coefficient *D*, we calculated the normalised LD measure *D*′ and the squared correlation coefficient *r* ^2^ (formulas in Table S2). The coefficient *D* captures the deviation of observed haplotype frequencies from those expected under linkage equilibrium. However, its magnitude is strongly dependent on allele frequencies and therefore not directly comparable across different locus pairs or populations. To account for this, *D*′ provides a frequency‐normalised measure that scales *D* relative to its theoretical maximum given the observed allele frequencies, allowing assessment of whether the deviation from equilibrium reflects near‐complete, partial, or weak disequilibrium independent of marginal allele distributions. In parallel, *r* ^2^ quantifies the statistical correlation between loci and is widely used as an indicator of how well one locus predicts another. Unlike *D* and *D*′, *r* ^2^ is directly interpretable in terms of information content and is particularly relevant for evaluating the extent of non‐independence among loci in population‐genetic or association contexts.

To reduce sparsity and facilitate statistical analyses, alleles with a frequency of less than 1% or with fewer than five occurrences were aggregated into a single ‘Other’ category before calculation of LD. Before plotting, we filtered the dataset by the *r* ^2^ values and omitted the ‘Other’ category. All haplotypes with an *r* ^2^ value below 0.01 (1%) were not included in the visualisations.

The use of all three measures (*D*, *D*′, and *r* ^2^) is especially important when analysing LD among more than two loci. However, calculating LD for a three‐locus system is uncommon and methodologically challenging, with a higher risk of estimation errors due to the rapid increase in haplotype states and uncertainty in their frequencies. Despite these limitations, *D* was assessed using a formula derived by Slatkin [13]. For calculation of the three‐locus *D*′, we used the formula proposed by Robinson et al. [14]. In contrast, no published formulas currently exist for *r* ^*2*^ in a three‐locus context. Therefore, an expression was constructed by extending the established two‐locus formulation to incorporate the additional locus (Table S2).

Because multi‐locus LD patterns can differ markedly from pairwise LD, relying on a single metric may obscure biologically meaningful aspects of haplotype structure. The coefficient *D* was used to quantify the direction and raw magnitude of deviations from expected haplotype frequencies, whereas *D′* provided a normalised measure that allowed comparisons across loci with markedly different allele‐frequency distributions—an important consideration for the highly polymorphic genes HLA‐A, HLA‐E, and HLA‐F. The *r* ^2^ statistic was included to quantify the predictive strength of association among loci within the three‐locus framework.

However, the calculations of *D*′ and *r* ^2^ can yield values outside the scope of definition of these parameters, especially when alleles with low frequencies are involved. Based on this limitation, we also calculated Beta as suggested by Gomez‐Raya et al. [15]. Beta (formula in Table S2) represents the proportion of total LD attributable to third‐order interactions in relation to second‐order components. It spans from 0 to 1, with 0 implying the absence of third‐order contributions, values greater than 0.5 suggesting that third‐order LD constitutes the majority of the signal, and 1 indicating full third‐order disequilibrium.

Together, these complementary metrics provide a more complete characterisation of the LD landscape and support a more robust interpretation of linkage patterns among the three loci.

## 3 Results

### 3.1 HLA‐F Primer Specificity

The optimised PCR conditions generated a single 3.9 kb product, as expected based on SNPcheck calculations and visualised using the IDEAL II DNA ladder from Blirt, with gel electrophoresis on a 1% agarose gel not showing any non‐specific bands. The selected primer pair, covering the entire HLA‐F gene, demonstrated consistent amplification efficiency and specificity across all tested DNA samples, including saliva‐derived DNA.

Initial performance testing with all pretyped DNA samples available at the time from the IHWG confirmed the accuracy of the chosen primer pair for HLA‐F amplification, with typing results generated using both Sanger sequencing and NGS.

### 3.2 Linkage Disequilibrium Between HLA‐F and HLA‐E

**FIGURE 2:**
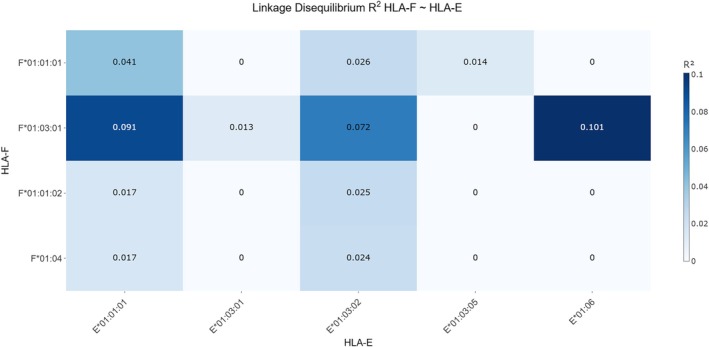
Heatmap of *r* ^2^ values between HLA‐F and HLA‐E alleles. The heatmap visualises pairwise LD between HLA‐F and HLA‐E alleles using the *r* ^2^ metric, which ranges from 0 to 1 and reflects the strength of statistical correlation between loci. Across all allele combinations, *r* ^2^ values remained low, falling between 0 and 0.1. Darker colours in the heatmap correspond to higher *r* ^2^ values, whereas lighter colours represent minimal or no detectable LD. This pattern indicates weak correlation and largely independent segregation of alleles at the two loci. Slightly elevated *r* ^2^ values were observed for specific haplotype HLA‐F*01:03:01 in combination with ‐E*01:06, but even in these cases the correlation did not exceed 0.1, highlighting the overall absence of strong LD between HLA‐F and HLA‐E.

Several haplotypes showed positive but modest disequilibrium like F*01:01:01 ~ E*01:01:01 with *D* = 0.047, *D*′ = 0.262, *r* ^2^ = 0.041 (Table 1 and Figure 2). These values indicate a slight enrichment of this haplotype relative to random expectation, although the low *r* ^2^ demonstrates limited predictive value of one allele for the other. Conversely, haplotype HLA‐F*01:03:01 in combination with ‐E*01:01:01 exhibited negative LD values (*D* = −0.059, *D*′ = −0.558, *r* ^2^ = 0.091), suggesting that these alleles co‐occur less frequently than expected by chance. The substantial magnitude of *D*′ in these comparisons reflects strong deviation from equilibrium after normalisation, whereas the *r* ^2^ values again indicate only weak correlation between the loci. LD patterns with HLA‐F*01:01:02 and F*01:04 were inconsistent in the context of *D*′ values, with most haplotypes showing low absolute *D* and *r* ^2^ values typically < 0.03. Haplotype HLA‐F*01:03:01 ~ E*01:03:02 (*D* = 0.050, *D*′ = 0.384, *r* ^2^ = 0.072) showed slightly elevated disequilibrium *D*′ but still did not reach levels indicative of meaningful co‐inheritance. The strongest LD signal was detected for the rare haplotype F*01:03:01 ~ E*01:06, which exhibited *D* = 0.019, *D*′ = 1.000, and *r* ^2^ = 0.101. Although the absolute *D* value remained small, the *D*′ value of 1.000 shows that within this allele pair the observed haplotype frequency reached the maximum possible value allowed by allele frequencies. All 31 cases of allele HLA‐E*01:06 were in combination with HLA‐F*01:03:01. Nevertheless, the *r* ^2^ remained low, suggesting that despite perfect normalised disequilibrium, this combination has limited utility for mutual prediction due to different allele frequencies.

**TABLE 1:** LD parameters *D* and *D*′ for HLA‐F and HLA‐E haplotypes.

Haplotype	*D*	*D*′
F*01:01:01~E*01:01:01	0.047	0.262
F*01:01:01~E*01:03:02	−0.036	−0.162
F*01:01:01~E*01:03:05	−0.006	−0.712
F*01:01:02~E*01:01:01	0.021	0.398
F*01:01:02~E*01:03:02	−0.024	−0.627
F*01:03:01~E*01:01:01	−0.059	−0.558
F*01:03:01~E*01:03:01	−0.012	−0.802
F*01:03:01~E*01:03:02	0.050	0.384
F*01:03:01~E*01:06	0.019	1.000
F*01:04~E*01:01:01	−0.009	−0.832
F*01:04~E*01:03:02	0.010	0.770

*Note:* The table summarises pairwise LD estimates for all observed HLA‐F~HLA‐E haplotypes. Overall, the absolute *D* values were small, indicating only modest deviations from random allele association. In contrast, several haplotypes displayed comparatively high normalised disequilibrium (*D*′), reflecting historical or population‐specific constraints on allele co‐occurrence even when absolute LD remained low. The strongest signal was observed for the haplotype F*01:03:01~E*01:06, which showed *D* = 0.019 and *D*′ = 1.000. A *D*′ value of 1.000 indicates that this allele pair reaches the maximum possible LD allowed by their frequencies, although the low *D* value suggests that the absolute deviation from random expectation remains small. In this dataset, all occurrences of allele HLA‐E*01:06 were observed in combination with HLA‐F*01:03:01; however, HLA‐F*01:03:01 is more frequent and also forms haplotypes with multiple other HLA‐E alleles, which reduces the absolute *D* value despite the perfect normalised disequilibrium.

Overall, the LD landscape between HLA‐F and HLA‐E is characterised by weak statistical association (low *r* ^2^ across haplotypes) but localised signals of high *D*′ in specific allele combinations, indicating historical or demographic influences without strong contemporary co‐segregation. These results demonstrate that HLA‐F and HLA‐E segregate largely independently, with only minor allele‐specific deviations from random association.

### 3.3 Linkage Disequilibrium Between HLA‐F and HLA‐A

**FIGURE 3:**
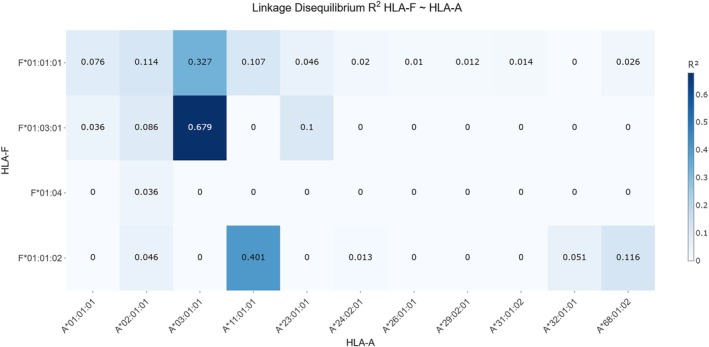
Heatmap of *r* ^2^ values between HLA‐F and HLA‐A alleles. The heatmap illustrates pairwise linkage disequilibrium between HLA‐F and HLA‐A alleles using the *r* ^2^ metric, which ranges from 0 to 1 and quantifies the strength of statistical correlation between loci. The heatmap uses darker colours to represent higher *r* ^2^ values, indicating stronger linkage disequilibrium, while lighter colours correspond to minimal or no detectable LD, providing a clear visual representation of the allele association landscape between HLA‐F and HLA‐A. In this dataset, *r* ^2^ values show a broader range, extending from 0 up to 0.679, indicating varying degrees of allele association. Haplotype HLA‐F*01:03:01~A*03:01:01 exhibits an elevated *r* ^2^ value of 0.679, reflecting strong linkage and a higher predictive correlation between these alleles. Other haplotypes demonstrate moderate to low *r* ^2^ values, suggesting weaker or limited correlation across most allele pairs.

The analysis of LD between HLA‐F and HLA‐A alleles revealed several haplotypes with notable associations (Table 2 and Figure 3). Among the observed haplotypes, HLA‐F*01:03:01 with ‐A*03:01:01 demonstrated the strongest LD, with a positive *D* value of 0.116, a near‐maximal *D*′ of 0.963 and a pronounced *r* ^2^ of 0.679. This suggests a strong and relatively frequent co‐occurrence of these alleles within the population, highlighting a significant haplotype block.

**TABLE 2:** Linkage disequilibrium parameters D and D′ for HLA‐F and HLA‐A haplotypes.

Haplotype	*D*	*D*′
F*01:01:01~A*01:01:01	0.045	0.985
F*01:01:01~A*02:01:01	0.071	0.768
F*01:01:01~A*03:01:01	−0.096	−0.956
F*01:01:01~A*11:01:01	−0.034	−0.980
F*01:01:01~A*23:01:01	−0.017	−0.870
F*01:01:01~A*24:02:01	0.019	0.646
F*01:01:01~A*26:01:01	0.009	0.782
F*01:01:01~A*29:02:01	0.008	1.000
F*01:01:01~A*31:01:02	0.009	1.000
F*01:01:01~A*68:01:02	−0.012	−0.714
F*01:01:02~A*02:01:01	−0.031	−0.944
F*01:01:02~A*11:01:01	0.045	0.985
F*01:01:02~A*24:02:01	−0.010	−0.988
F*01:01:02~A*32:01:01	0.013	0.430
F*01:01:02~A*68:01:02	0.017	0.783
F*01:03:01~A*01:01:01	−0.026	−0.974
F*01:03:01~A*02:01:01	−0.052	−0.954
F*01:03:01~A*03:01:01	0.116	0.963
F*01:03:01~A*23:01:01	0.021	0.892
F*01:04~A*02:01:01	0.012	0.851

*Note:* The table summarises pairwise LD estimates for observed HLA‐F~HLA‐A haplotypes. Overall, absolute *D* values ranged from small to moderate, indicating varying degrees of deviation from random allele association across different haplotypes. Several haplotypes displayed high normalised disequilibrium (*D*′), often approaching or reaching values near 1.0, which reflects strong historical or population‐specific constraints on allele co‐occurrence despite sometimes modest absolute LD. Notably, the haplotype F*01:03:01~A*03:01:01 exhibited one of the highest linkage signals, with *D* = 0.116 and *D*′ = 0.963. Other important haplotypes, such as F*01:01:02~A*11:01:01 (*D* = 0.045, *D*′ = 0.985) and F*01:01:01~A*02:01:01 (*D* = 0.071, *D*′ = 0.768), showed moderate absolute *D* values paired with high *D*′, consistent with relatively strong allelic association.

Similarly, F*01:01:02 ~ A*11:01:01 showed substantial LD with *D* = 0.045, *D*′ = 0.985 and *r* ^2^ = 0.401, indicating a strong normalised disequilibrium and moderate correlation between these alleles. Other notable haplotypes include F*01:01:01 ~ A*02:01:01 (*D* = 0.071, *D*′ = 0.768, *r* ^2^ = 0.114) and F*01:01:01 ~ A*01:01:01 (*D* = 0.045, *D*′ = 0.985, *r* ^2^ = 0.076), which both show moderate levels of LD and allele association. The high *D*′ values indicate that these allele pairs exhibit strong normalised LD relative to their allele frequencies, meaning recombination between them is rare. However, the low *r* ^2^ values reflect that the correlation between their allele frequencies is modest, often due to differences in allele frequencies or the presence of these alleles in multiple haplotypic backgrounds, reducing the predictive power of one allele for the other.

Conversely, some haplotypes such as F*01:01:01 ~ A*03:01:01 exhibited a negative *D* (−0.096) and *D*′ (−0.956), but with a relatively high *r* ^2^ (0.327), indicating an inverse association yet significant predictability.

Overall, these results demonstrate a pattern of moderate to strong LD between specific HLA‐F and HLA‐A alleles, with several haplotypes showing high normalised disequilibrium (*D*′ close to ±1), reflecting constrained recombination or population‐specific allele pairing. However, the range of *r* ^2^ values suggests varying degrees of predictability in these associations, with F*01:03:01~A*03:01:01 being the most strongly linked pair in this dataset.

### 3.4 Linkage Disequilibrium Between HLA‐A and HLA‐E

**FIGURE 4:**
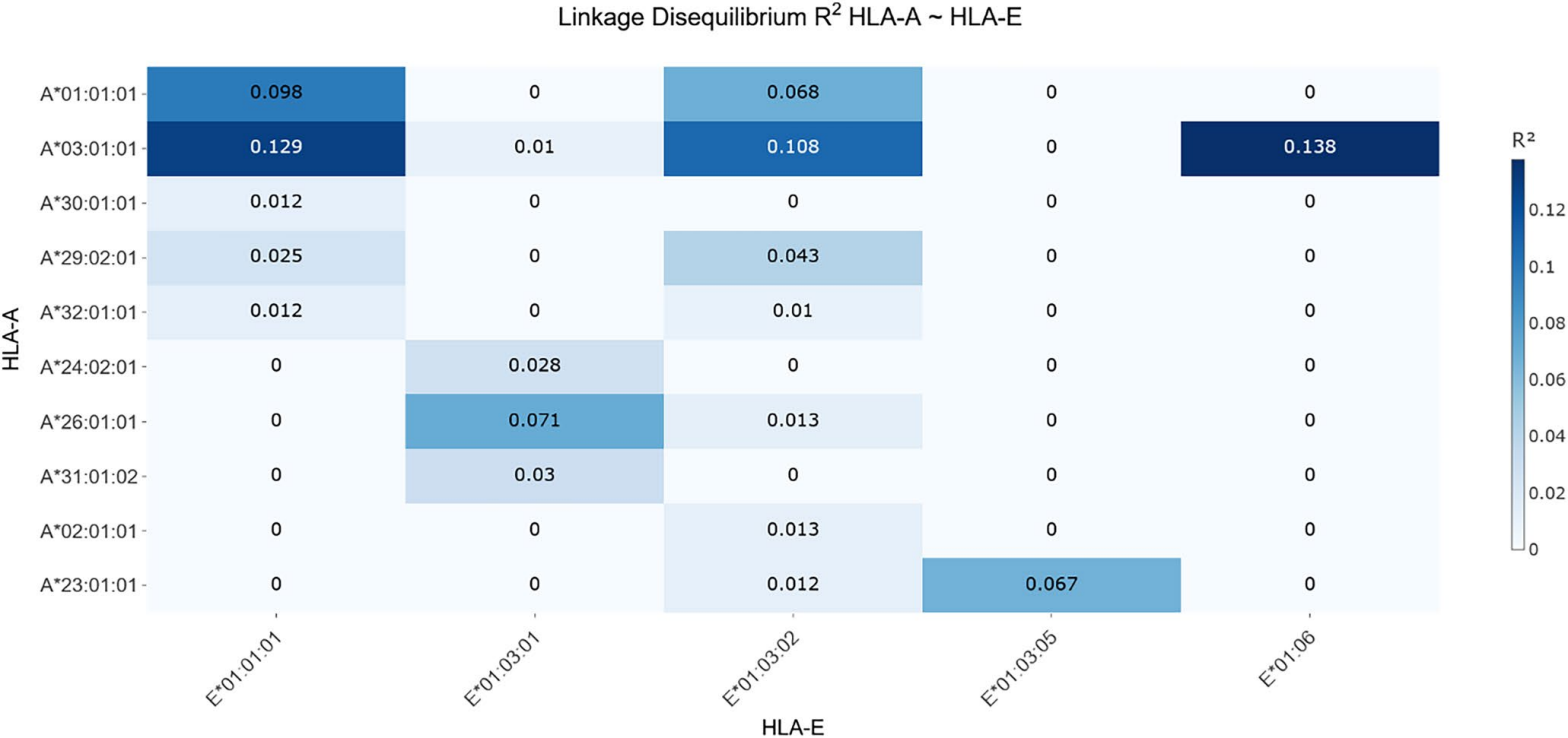
Heatmap of *r* ^2^ values between HLA‐A and HLA‐E alleles. The heatmap illustrates pairwise LD between HLA‐A and HLA‐E alleles using the *r* ^2^ metric, which ranges from 0 to 1 and quantifies the strength of statistical correlation between loci. The heatmap uses darker colours to represent higher *r* ^2^ values, indicating stronger LD, while lighter colours correspond to minimal or no detectable LD, providing a clear visual representation of the allele association landscape between HLA‐F and HLA‐A. In this dataset, *r* ^2^ values were limited and show a narrow range, extending from 0 up to 0.138.

The linkage between HLA‐A and HLA‐E (Table 3, Figure 4) was more pronounced than the detected linkages between HLA‐F and HLA‐E. HLA‐A*03:01:01 seems to be in repulsion with HLA‐E*01:01:01 (*D*′ = −0.774; *r* ^2^ = 0.129) while in strong linkage with HLA‐E*01:06 (*D*′ = 1.000; *r* ^2^ = 0.138). HLA‐E*01:03:02 is only in moderate linkage with HLA‐A*03:01:01 (*D*′ = 0.552; *r* ^2^ = 0.108). The combination of other HLA‐A and HLA‐E genotypes with high frequencies in the cohort displays high *D*′ values while the corresponding *r* ^2^ values are relatively low.

**TABLE 3:** LD parameters *D* and *D*′ for HLA‐A and HLA‐E haplotypes.

Haplotype	*D*	*D*′
A*01:01:01~E*01:01:01	0.054	0.867
A*01:01:01~E*01:03:02	−0.042	−0.928
A*02:01:01~E*01:03:02	0.024	0.125
A*03:01:01~E*01:01:01	−0.064	−0.774
A*03:01:01~E*01:03:01	−0.010	−0.844
A*03:01:01~E*01:03:02	0.055	0.552
A*03:01:01~E*01:06	0.020	1.000
A*23:01:01~E*01:03:02	−0.009	−0.916
A*23:01:01~E*01:03:05	0.005	0.391
A*24:02:01~E*01:03:01	0.013	0.181
A*26:01:01~E*01:03:01	0.013	0.412
A*26:01:01~E*01:03:02	−0.010	−0.877
A*29:02:01~E*01:01:01	−0.012	−0.904
A*29:02:01~E*01:03:02	0.015	0.921
A*30:01:01~E*01:01:01	0.007	0.960
A*31:01:02~E*01:03:01	0.008	0.294
A*32:01:01~E*01:01:01	0.010	0.649
A*32:01:01~E*01:03:02	−0.009	−0.750

*Note:* The table summarises pairwise LD estimates for observed HLA‐A~HLA‐E haplotypes. The absolute *D* values were generally small, ranging from approximately −0.064 to 0.055, indicating only modest deviations from random allele association. Several haplotypes displayed high normalised LD *D*′, positive and negative, often approaching values near ±1.000. Notably, the haplotype A*03:01:01~E*01:06 reaches the highest *D*′ of 1.000, despite having a low absolute *D* value of 0.020, indicating strong normalised linkage relative to allele frequencies but only a modest deviation from random association.

### 3.5 LD Between HLA‐A, HLA‐E, and HLA‐F

LD analyses across the three‐locus combinations revealed moderate associations between several allele sets (Table 4). The highest LD was observed for the haplotype HLA‐F*01:03:01 ~ A*03:01:01 ~ E*01:01:01, which showed an *r* ^2^ of 0.387, indicating a substantial degree of non‐random allelic association. Similarly elevated LD values were detected for HLA‐F*01:03:01 ~ A*03:01:01 ~ E*01:03:02 (*r* ^2^ = 0.345) and HLA‐F*01:03:01 ~ A*03:01:01 ~ E*01:06 (*r* ^2^ = 0.362).

**TABLE 4:** Graphical overview of linkage disequilibrium (LD) between HLA‐A, HLA‐E and HLA‐F.

Haplotype	*D*	*r* ^2^	*β*
F*01:01:01~A*03:01:01~E*01:01:01	0.036	0.185	0.148
F*01:01:01~A*03:01:01~E*01:03:02	−0.033	0.172	0.149
F*01:01:01~A*24:02:01~E*01:01:01	−0.010	0.020	0.112
F*01:01:01~A*29:02:01~E*01:03:02	0.006	0.028	0.089
F*01:01:02~A*11:01:01~E*01:01:01	0.007	0.041	0.088
F*01:01:02~A*11:01:01~E*01:03:02	−0.006	0.035	0.074
F*01:01:02~A*68:01:02~E*01:01:01	0.005	0.035	0.103
F*01:01:02~A*68:01:02~E*01:03:02	−0.004	0.024	0.078
F*01:03:01~A*03:01:01~E*01:01:01	−0.044	0.387	0.154
F*01:03:01~A*03:01:01~E*01:03:01	−0.006	0.027	0.043
F*01:03:01~A*03:01:01~E*01:03:02	0.039	0.345	0.150
F*01:03:01~A*03:01:01~E*01:06	0.013	0.362	0.077
F*01:03:01~A*23:01:01~E*01:01:01	0.006	0.034	0.067
F*01:03:01~A*23:01:01~E*01:03:02	−0.008	0.062	0.089
F*01:03:01~A*23:01:01~E*01:03:05	0.004	0.279	0.119
F*01:04~A*02:01:01~E*01:03:02	0.006	0.040	0.114

*Note:* The table shows the values for *D*, *r* ^2^ and *β*. *D* indicates the direction and magnitude of deviation from expected haplotype frequencies in linkage disequilibrium. *D* > 0 indicates the haplotype occurs more often than expected and *D* < 0 indicates the haplotype occurs less often than expected. *r* ^2^ reflects how well one allele predicts the other, representing the correlation between loci. *r* ^2^ ranges from 0 to 1 and higher values indicate stronger correlation or association between the alleles. *β* quantifies the contribution of third‐order LD relative to second‐order LD, reflecting the interdependence between these coefficients. Its values range from 0 to 1. *β* = 0 denotes that all LD is attributable to second‐order interactions, values > 0.5 indicate that third‐order components predominate, and *β* = 1 signifies that LD is entirely third‐order in origin. D' is not reported in the table because it yielded values outside the defined range of this parameter, which occurred when low‐frequency alleles were involved.

The value of *D* varied among haplotypes, reflecting differences in whether alleles occurred together more or less frequently than expected under random association. Notably, the strongest LD signals, as indicated by *r* ^2^, occurred both for haplotypes with positive *D* (e.g., F*01:03:01~A*03:01:01~E*01:03:02) and negative *D* (e.g., F*01:03:01~A*03:01:01~E*01:01:01), underscoring that the magnitude rather than the direction of *D* was most relevant for the strength of association. Taking Beta into account as a measure of the relative contribution of third‐order LD, the corresponding values below *β* = 0.2 suggest that the observed associations are driven predominantly by pairwise LD.

### 3.6 HLA‐F Allele Frequency

We detected 14 HLA‐F alleles, with F*01:01:01:01 being the most frequent allele (21.23% of our cohort), closely followed by F*01:03:01:01 (20.65%) (Table 5). In addition, the alleles F*01:01:01:09 (20.19%) and F*01:01:01:08 (18.43%) were found quite often in the studied population. HLA‐F*01:04 could only be observed in 19 individuals and F*01:05 or F*01:06 could not be found in even one case. Grouping the different intronic variants makes a total amount of 66.54% for allelic group HLA‐F*01:01:01 and 11.15% for HLA‐F*01:01:02 in general.

**TABLE 5:** Proportion of HLA‐F alleles (four fields) in the analysed cohort of 763 samples, *n* = number of alleles, frequency is given as proportion.

Allele	*n*	Frequency	Homozygous
F*01:01:01:01	326	0.213	31
F*01:03:01:01	317	0.206	33
F*01:01:01:09	310	0.201	30
F*01:01:01:08	283	0.184	29
F*01:01:02:04	143	0.093	7
F*01:01:01:05	79	0.051	2
F*01:01:01:11	25	0.016	0
F*01:01:02:07	24	0.015	1
F*01:04	19	0.012	0
F*01:01:02:03	4	0.002	0

### 3.7 Detection of Variants in HLA‐F by NGS and ONT

Sequence variants detected by NGS were compared with variants detected by ONT to confirm the result. In conclusion, the present report describes nine new HLA‐F alleles (Table 6) that have been identified in high‐throughput HLA typing of 673 newly registered stem cell donors at the Stefan Morsch Stiftung in 2017 and 2018. The large number of single nucleotide polymorphisms found in the exon region of the samples, partly silent and partly causing an exchange in the amino acid, shows that HLA‐F has a higher haplotype variability than previously described by the IPD‐IMGT/HLA Database. Some of the new alleles were observed more than once in the analysed samples, showing that they are not necessarily rare even though they are newly identified. It rather shows that the locus has a higher allelic polymorphism than described in previously published works and that a closer examination of the non‐classical gene could generate new results.

**TABLE 6:** Description of the validated new allele sequences of HLA‐F.

Sample ID	Accession number	IPD‐IMGT/HLA database submission	Mismatch gDNA	Amino acid change	Exon	IPD‐IMGT/HLA database nomenclature
A‐552889	MZ274324	HWS10061534	c.310 C>T	F60F	2	F*01:03:02
A‐577532	ON615425	HWS10061784	c.974T>C	L200P	4	F*01:10
A‐595748	ON866546	HWS10062142	c.985G>A	D204N	4	F*01:09
A‐590131	MZ274327	HWS10061540	c.985G>A	D204N	4	F*01:09
A‐556389	MZ274326	HWS10061538	c.1590 C>T, c.2411 A>G	P211P S272P	4 4	F*01:01:09
A‐556214	OM519330	HWS10061536	c.1723 C>A	P256T	4	F*01:08
A‐603803	MZ274328	HWS10063507	c.1777G>A	E274K	4	F*01:07
A‐577738	ON615426	HWS10061786	c.2070G>A	A325T	6	F*01:11

The HLA‐F sequences were submitted to the IPD‐IMGT/HLA Database and have been officially assigned by the WHO Nomenclature Committee for Factors of the HLA System in November 2021 and June 2022. This follows the agreed policy that, subject to the conditions stated in the most recent Nomenclature Report [16], names will be assigned to new sequences as they are identified. Lists of such new names will be published in the following WHO Nomenclature Report.

Additional sequence extensions for the newly added alleles F*01:01:04, F*01:01:05, F*01:01:06 and F*01:01:07 were submitted to the IPD‐IMGT/HLA Database.

As observed, the majority of the identified variations are located within exon 4 of the HLA‐F gene, while there was only one novel detected amino acid change in exon 2 and one in exon 6. This concentration of variants in a single exon may have significant implications, as exon 4 encodes a critical region involved in the molecule's structural integrity and function. Variations here could potentially affect peptide binding, receptor interactions, or protein stability, thereby influencing immune recognition and response.

## 4 Discussion

Here, we present a long‐range PCR sequencing technique for genotyping HLA‐F using the MiSeq platform. A similar approach was published in 2016 by Lima et al. [17], using a Nextera XT Sample Preparation Kit. We used a modified way for the library preparation to circumvent the bias in template fragmentation. Apart from that, we also used alternative amplification primers which have been evaluated with IHWG cell lines, pretyped for HLA‐F, to show both the specificity for HLA‐F and the lack of interference with other HLA class I genes. Due to the limited number of well‐characterised HLA‐F reference samples, no additional cell lines with alternative allele constellations were available for testing at the time. Consequently, it cannot be excluded that some rare alleles currently present in public databases were not captured by the initial validation set. However, the functionality and specificity of the used primer pair were confirmed across all available cell lines, supporting their robustness within the context of the most prevalent alleles.

The aim of this study was to genotype HLA‐F alleles in a healthy cohort of 763 volunteer blood donors, residing in Germany. The lack of data on the distribution of non‐classical HLA alleles in healthy individuals represents a significant limitation for gene‐disease association studies, for understanding the influence of HLA‐F products in solid organ and stem cell transplantation, and for immunogenetic research in general. To date, few studies have investigated the allelic variability of HLA‐F, and even fewer have reported four‐field allele frequencies [18]. Although HLA‐F is thought to exhibit few polymorphisms, gene‐disease association studies are lacking.

LD, the non‐random association of alleles at different loci, has been reported between HLA‐F and various class I and II genes across different populations. In our study of 763 healthy potential stem cell donors residing in Germany, we observed LD patterns consistent with those previously described, while also uncovering novel insights enabled by high‐resolution three‐field genotyping.

In previous studies, Manvailer et al. identified a strong LD between HLA‐F*01:01 and HLA‐A*02 in a Euro‐Brazilian cohort [19]. We confirmed this association in our dataset; however, three‐field resolution revealed a more precise relationship.

Further, consistent with findings from Pan et al. [8], we observed strong LD between HLA‐F*01:03, HLA‐A*03, and HLA‐E*01:03, a haplotype combination previously described in Mainland Chinese populations. In our dataset, the extended haplotype F*01:03:01 ~ A*03:01:01 ~ E*01:03:02 showed a negative *D* value of −0.033, but an elevated *r* ^2^ of 0.172 and *β* of 0.149, indicating non‐random association among these alleles in line with Pan et al.'s observations, though with somewhat lower LD strength—likely reflecting population differences or greater allelic resolution in our study. Importantly, our high‐resolution genotyping revealed additional patterns not previously reported. Beyond the association of HLA‐F*01:03 with HLA‐E*01:03:02, there is also displayed LD with HLA‐F*01:04, forming the haplotype F*01:04 ~ E*01:03:02, characterised by *D* = 0.010 and *D*′ = 0.770, suggesting a strong normalised association despite modest absolute deviation. This additional combination was not reported by Pan et al. and may reflect either broader population diversity or improved allele‐level resolution afforded by our dataset. Our higher‐resolution genotyping revealed a more nuanced LD structure than reported by Pan et al. Specifically, HLA‐F*01:03:01 also showed notable (negative) LD with HLA‐E*01:03:01 (*D* = −0.012, *D*′ = −0.802) and, most prominently, with HLA‐E*01:06, which displayed the strongest association in our dataset (*D* = 0.019, *D*′ = 1.000). These additional F*01:03‐linked haplotypes were not observed in the earlier study and suggest that the genomic context of HLA‐F*01:03 extends beyond the canonical A*03 ~ E*01:03 combination. Together, these findings indicate that while the key LD relationships involving HLA‐F*01:03 are consistent with previous work, the full extent of its linkage network appears broader and more diverse when analysed at allelic high resolution.

Analysis of LD between HLA‐F and its neighbouring loci HLA‐A and HLA‐E revealed distinct patterns depending on the locus pair. For HLA‐F with HLA‐E, we observed high *D*′ values, including one allele combination reaching *D*′ = 1.000, while all *r* ^2^ values remained low (maximum *r* ^2^ = 0.100). This discrepancy reflects the fundamental difference between the two measures: *D*′ captures the absence of historical recombination, whereas *r* ^2^ quantifies predictive correlation and is strongly constrained by allele‐frequency imbalance. Thus, even when *D*′ indicates that specific haplotypes have been preserved, *r* ^2^ can remain low if the alleles involved segregate at markedly different frequencies. A similar but somewhat stronger pattern was observed for HLA‐F with HLA‐A. Several allele pairs exhibited very high *D*′ values (up to *D*′ = 1.000), yet the corresponding *r* ^2^ values, although higher than those for HLA‐F~HLA‐E, remained substantially lower than expected based on *D*′ alone. This again illustrates that *D*′ is sensitive to the presence or absence of rare haplotypes, whereas *r* ^2^ requires both historical conservation and compatible allele frequencies to yield strong correlations. The higher *r* ^2^ values for HLA‐F~HLA‐A likely reflect the closer physical proximity of these loci, which may have reduced recombination over evolutionary time compared with the more distant HLA‐F~HLA‐E pair. In summary, the combination of high *D*′ but low *r* ^2^ observed for HLA‐F~HLA‐E indicates limited historical recombination but weak allelic covariance, whereas HLA‐F~HLA‐A shows stronger LD in both metrics, likely due to shorter genomic distance and a more conserved haplotypic configuration. These results underscore the necessity of evaluating both *D*′ and *r* ^2^ when interpreting LD, as each metric provides distinct and complementary insights into the evolutionary and structural relationships among HLA loci.

The application of complementary LD measures highlights the complexity of linkage relationships among HLA‐A, HLA‐E, and HLA‐F. The three‐locus *D* and *D*′ values revealed that several allele combinations show deviations from random assortment, although the magnitude of absolute disequilibrium varied considerably across the loci. High *D*′ values, in particular, indicate that for some haplotypes, the observed frequencies approach the maximum possible disequilibrium allowed by their allele frequencies, suggesting historical constraints on recombination or long‐standing population structure. However, *r* ^2^ values remained generally low, underscoring that even when normalised LD is high, the predictive correlation between alleles across the three loci is limited. This discrepancy between *D*′ and *r* ^2^ reflects the well‐known influence of allele‐frequency differences and highlights why multi‐metric LD assessment is essential for interpreting HLA haplotype structure. Since the calculations of *D*′ yielded values outside the defined scope of this parameter in some cases due to the involvement of low‐frequency alleles, β was calculated instead, as it remains valid when low allele frequencies distort *D*' and provides a more accurate characterisation of the underlying disequilibrium structure by accounting for higher‐order LD components.

Collectively, the results demonstrate that the three‐locus LD landscape is shaped by both strong historical linkage signals and ongoing reshuffling of alleles, producing patterns in which some haplotypes are tightly conserved while others show substantial independence. These findings emphasise the importance of considering multi‐locus LD when studying HLA evolution, population diversity, or immunogenetic associations—while also recognising that three‐locus LD analyses are rarely performed and methodologically challenging due to the scarcity of established formulas in the literature. Overall, several three‐locus combinations involving F*01:03:01 and A*03:01:01 exhibited particularly strong LD, suggesting the presence of conserved haplotypic structures within this genomic region. Most haplotypes included the allele A*03:01:01, which reflects its high frequency (*n* = 595) in the dataset. Consequently, LD patterns are largely driven by haplotypes carrying this allele.

Although the absolute *D* values were small (−0.04 to +0.04), this is expected for multi‐allelic HLA loci due to frequency constraints. The magnitude of *D* is mathematically limited by the marginal allele frequencies, meaning that even substantial LD produces only small absolute *D* values. Nevertheless, the corresponding *r* ^2^ values indicate moderate to strong linkage disequilibrium for specific three loci combinations like HLA‐F*01:03:01~A*03:01:01~E*01:01:01 (*r* ^2^ = 0.387), HLA‐F*01:03:01~A*03:01:01~E*01:03:02 (*r* ^2^ = 0.345), and HLA‐F*01:03:01~A*03:01:01~E*01:06 (*r* ^2^ = 0.362). Taken the information provided by *β* into consideration, the results indicate that despite high linkage most of the observed three‐locus LDs are mainly driven by their pairwise components.

In our study, LD was estimated from partly unphased genotype data. This approach is supported by the findings of Hui and Burt [20], who showed that LD estimators derived from unphased genotypes can recover true LD levels under Hardy–Weinberg equilibrium (HWE), despite the absence of phase information. Their simulations demonstrated that both phased and unphased estimators exhibit finite‐sample upward bias, but that this bias is analytically predictable and can be corrected, yielding unbiased LD estimates in expectation. A key difference, however, is that unphased estimators consistently display higher variance than phased estimators, reflecting reduced precision. Given our large sample size (*n* = 763), this loss of precision is expected to be minimal, as estimator variance decreases substantially with increasing sample size. Allele‐frequency variation in our dataset—including low‐frequency and common alleles—may influence the distribution of LD estimates, but under the HWE‐based likelihood framework, such frequency differences do not introduce systematic bias into the estimation process. Therefore, although our LD estimates should be interpreted with awareness of the inherently higher variance associated with unphased data, the combination of a large sample size and appropriate statistical modelling supports the reliability and robustness of our LD inferences.

Regarding allelic distribution, our study confirms and expands upon previous observations. Closa [21] reported HLA‐F*01:01 at a frequency of 84.90% and ‐F*01:03 at 14.06% in a Catalonian cohort, based on two‐field resolution. Using four‐field typing, we were able to resolve HLA‐F*01:01 into seven distinct alleles, with ‐F*01:01:01:08 (26.04%) being the most frequent, followed by ‐F*01:01:01:01 (23.44%) and ‐F*01:01:01:09 (15.51%). HLA‐F*01:03:01:01 was detected in 9.90% of our cohort, slightly lower than the frequency reported by Closa et al. Interestingly, HLA‐F*01:04, which was not observed in the Catalonian study, was found in 19 of our 763 samples. This discrepancy is likely not due to population‐specific allele frequencies, as the Catalonian samples originated from unrelated blood donors, but rather a consequence of the small sample size (*n* = 96) and the low frequency of this allele.

Taken together, our findings not only support previously described LD patterns but also provide more detailed haplotype definitions due to the increased resolution of four‐field sequencing. This enhanced genotyping approach improves our understanding of HLA‐F allele distribution and linkage patterns, and lays the groundwork for future studies on their functional implications in transplantation, autoimmunity, and disease association.

In general, the present study clearly shows that HLA‐F is not as conserved at the protein level as thought a decade ago. Lima et al. [17] reported in 2016 that only 30 coding alleles were detected, encoding four different HLA‐F full‐length protein molecules. When we started this study in 2018, 56 coding alleles were reported, encoding 11 different protein molecules. To date (as of IPD‐IMGT/HLA Database Release 3.62.0, 2025‐10‐08), 126 coding alleles have been registered, encoding 30 different HLA‐F protein molecules. And as sequencing technologies advance, the number of registered alleles is expected to increase, reflecting the increasing interest in non‐classical HLA genes. This expanding focus promises to deepen our understanding of HLA‐F's complex role in immune response and its clinical relevance.

## Author Contributions

Nina Grünen performed the experiments and did the data analysis as part of her Bachelor degree, and prepared the manuscript. Liane Hey did the NGS training. Yannik Busch was responsible for Nanopore sequencing, IPD‐IMGT/HLA Database submission and Linkage Disequilibrium (LD) calculations, including graphical visualisation. Prof. Dr. Anne Schweizer was co‐supervisor. Dr. Marco Schäfer as HLA‐lab director of the Stefan Morsch Stiftung provided the samples. Dr. Wolfgang Peter as head of R&D at the Stefan Morsch Stiftung did the concept of work and supervision. Ilias Doxiades performed critical reading.

## Ethics Statement

Blood samples used in this study were collected as part of routine stem cell donor registration by Stefan Morsch Stiftung (Birkenfeld, Germany), with all donors providing informed consent for HLA typing and for the use of their samples and data for research purposes. As the samples are fully anonymised prior to analysis and used in accordance with the donors' consent, no additional ethical approval was required.

## Conflicts of Interest

The authors declare no conflicts of interest.

## Supporting information


**Table S1:** Used IHWG pretyped DNA samples for performance testing.

**Table S2:** Formulas to calculate linkage disequilibrium (LD).

## Data Availability

The data that support the findings of this study are available on request from the corresponding author. The data are not publicly available due to privacy or ethical restrictions.
