# Supplementary material for: HLA‐F: A Non‐Classical Gene With Growing Interest
Source: HLA. 2026 Jan 15;107(1):e70547. doi: 10.1111/tan.70547 (PMC12805605; doi:10.1111/tan.70547)
Supplement: Supplementary file 1 — Table S1: Used IHWG pretyped DNA samples for performance testing. [file TAN-107-e70547-s001.docx]

**Supplement Table 1: Used IHWG pretyped DNA samples for performance testing**

| IHW # | name | HLA-F alleles |
| --- | --- | --- |
| IHW09015 | WT24 | 01:01:01:01 |
| IHW09062 | WDV | 01:01:01:01 |
| IHW09052 | DBB | 01:01:01:08 |
| IHW09040 | BM15 | 01:01:01:09 |
| IHW09107 | LKT3 | 01:01:01:09 |
| IHW01143 | 1408-1014 | 01:01:01:10 |
| IHW09302 | SSTO | 01:01:02:06 |
| IHW09318 | PGF | 01:03:01:01 |
| IHW01173 | 1416-1186 | 01:03:01:02 |
| IHW01141 | 1408-1012 | 01:01:01:03, 01:01:01:07 |
| IHW01175 | 1416-1188 | 01:01:01:04, 01:01:02:03 |
| IHW01181 | 1416-1194 | 01:01:02:03, 01:04 |
| IHW01182 | 1416-1195 | 01:03:01:01, 01:04 |
| IHW01184 | 1416-1197 | 01:01:01:04, 01:03:01:02 |
