# Supplementary material for: HLA‐F: A Non‐Classical Gene With Growing Interest
Source: HLA. 2026 Jan 15;107(1):e70547. doi: 10.1111/tan.70547 (PMC12805605; doi:10.1111/tan.70547)
Supplement: Supplementary file 2 — Table S2: Formulas to calculate linkage disequilibrium (LD). [file TAN-107-e70547-s002.docx]

**Supplement Table 2: Formulas to calculate Linkage Disequilibrium (LD)**

The **allele frequencies** are calculated as:

P(A) = $\frac{nA}{N}$ , P(B) = $\frac{nB}{N}$ , P(C) = $\frac{nC}{N}$

- nA, nB, nC = counts of individuals carrying each allele independently
- N = total number of chromosomes (alleles) sampled

**Linkage Disequilibrium Coefficient (D)**

The **linkage disequilibrium coefficient (D)** measures the deviation of the observed haplotype frequency from the expected frequency under independence (Hill und Weir 1988):

D = P(ABC) - P(A) x P(BC) - P(B) x P(AC) - P(C) x P(AB) + 2*(P(A) x P(B) x P(C))

- (D > 0) indicates the haplotype occurs more often than expected.
- (D < 0) indicates the haplotype occurs less often than expected.

**Normalized Linkage Disequilibrium (D')**

The **standardized coefficient (D')** rescales (D) to the maximum possible value given allele frequencies:

**D' =** $\frac{\boldsymbol{D}}{\boldsymbol{Dmax}}$

Where D_max_ is the theoretical maximum or minimum possible value:

D_max_ = $\left\{ \frac{\min\left[ P\left( A \right)\left( 1-P\left( B \right) \right), P\left( B \right)\left( 1-P\left( A \right) \right) \right], if D > O}{\min\left[ P\left( A \right)P\left( B \right), P\left( \left( 1-A \right)\left( 1-B \right) \right) \right], if D < 0} \right.$

D' ranges from -1 to +1, allowing comparison across alleles with different frequencies.

**Squared Correlation r²**

The **r-squared (r²)** value quantifies the proportion of variance in one allele that is explained by the others:

r² = $\frac{D^{2}}{P\left( A \right)\left( 1-P\left( A \right) \right)x P\left( B \right)\left( 1-P\left( B \right) \right) x P(C)(1-P\left( C \right))}$

- r² ranges from 0 to 1.

Higher values indicate stronger correlation or association between the alleles

**Normalized third order Linkage Disequilibrium (β)**

The relative value of third order disequilibrium versus second order disequilibrium is defined by (Gomez-Raya et al. 2018):

$$\beta_{ABC}=\frac{abs\left( D_{ABC} \right)}{abs\left( D_{ABC} \right)+abs\left( D_{AB} \right)+abs\left( D_{AC} \right)+abs\left( D_{BC} \right)}$$

β_ABC_ ranges from 0 to 1, where 0 indicates that LD is second order, values over 0.5 indicate that LD is mostly third order and 1 that all LD is third order.

Literature:

Gomez-Raya, Luis; Silio, Luis; Rauw, Wendy M.; Gracia-Cortés, Luis Alberto; Rodríguez, Carmen (2018): Extent of third-order linkage disequilibrium in a composite line of Iberian pigs. In: *BMC genetics* 19 (1), S. 60. DOI: 10.1186/s12863-018-0661-4.

Hill, W. G.; Weir, B. S. (1988): Variances and covariances of squared linkage disequilibria in finite populations. In: *Theoretical population biology* 33 (1), S. 54–78. DOI: 10.1016/0040-5809(88)90004-4.
